# Statement from the President of the *International Association for Child and Adolescent Psychiatry and Allied Professions*

**DOI:** 10.1186/1753-2000-1-2

**Published:** 2007-06-26

**Authors:** Per-Anders Rydelius

**Affiliations:** 1 Department of Woman and Child Health, Child and Adolescent Psychiatric Unit, Karolinska Institutet, Stockholm, Sweden

## Editorial

The new BioMed Central online journal on *Child and Adolescent Psychiatry and Mental Health *is a very welcome contribution to the field of child and adolescent psychiatry and an important step forward to spread scientific information related to issues of child and adolescent mental health.

For many years the *Acta Paedopsychiatrica *(founded by Professor Tramer in Switzerland and published as the 'Z*eitschrift für Kinderpsychiatrie' *1934–1952 and then as '*Acta Paedopsychiatrica'*, 1953–1994) was the "The Publishing Organ for the Official Bulletin of the IACAPAP, the International Association for Child and Adolescent Psychiatry and Allied Professions". As an official organ, the *Acta Paedopsychiatrica *served many purposes. It was a "multilingual" journal where papers in English, French and German were published. The abstracts were written in four languages including Spanish. The information from IACAPAP included proceedings from the Association's international congresses and other news as well. Thematic issues covering topics of special interests were published on a regular basis. One of these, the *Acta Paedopsychiatrica *vol 35. Fasc. 4–8 p. 97–248 from 1968, presented an overview of the research and current opinions on Autism Infantum in those days. Of special interest is that Leo Kanner was the author of the first chapter presenting the Kanner Syndrome while Hans Asperger was the author of the second chapter describing his syndrome.

Today, the IACAPAP Bulletin, accessible on the IACAPAP web page [1], gives the official IACAPAP information while an equally easily available scientific journal is lacking. For this reason, the new online journal "*Child and Adolescent Psychiatry and Mental Health*" will be of special importance for scientific purposes in our field. Being an open access journal, and therefore freely available via the internet, it will give child and adolescent psychiatrists and mental health workers all over the world unique opportunities and without the barrier of subscription rates to follow the frontiers of knowledge and experiences in our disciplines. This is even more important for clinicians and researchers in developing countries where economy may restrict their chances to subscribe for scientific journals.

Stockholm and Karolinska Institutet May 2007
